# Bis{(*R*)-*N*-[(*R*)-2-benz­yloxy-1-(4-*tert*-butyl­phen­yl)eth­yl]-2-methyl­propane-2-sulfinamide} monohydrate

**DOI:** 10.1107/S1600536814004589

**Published:** 2014-03-08

**Authors:** Charlotte L. Humes, Tyler J. Banker, Stephanie C. M. Dorn, James P. Shanahan, William W. Brennessel, Daniel J. Weix

**Affiliations:** a Department of Chemistry, 120 Trustee Road, 412 Hutchison Hall, University of Rochester, Rochester, NY 14627, USA

## Abstract

The asymmetric unit of the title compound, 2C_23_H_33_NO_2_S·H_2_O, contains one organic mol­ecule in a general position and one co-crystallized water mol­ecule on a crystallographic twofold axis. Each water mol­ecule serves as a hydrogen-bond donor to a pair of S=O acceptors on symmetry-related mol­ecules. Thus, each trio of mol­ecules forms one title formula unit. These groupings are further connected along [010] *via* weak non-classical C—H⋯O hydrogen bonds.

## Related literature   

For a general method to synthesize the Grignard reagent used in the reaction that generated the title material, see: Tilstam & Weinmann (2002 ▶). For in-depth discussions on methods to synthesize the precursor to the title mol­ecule from 2-butene-1,4-diol, see: Evans *et al.* (2008 ▶); Tang *et al.* (2001 ▶). For the importance of 1,2-amino­alcohols, see: Bergmeier (2000 ▶). For methods used to determine the absolute configuration, see: Flack (1983 ▶); Parsons & Flack (2004 ▶); Parsons *et al.* (2013 ▶). For a description of the Cambridge Structural Database, see: Allen (2002 ▶).
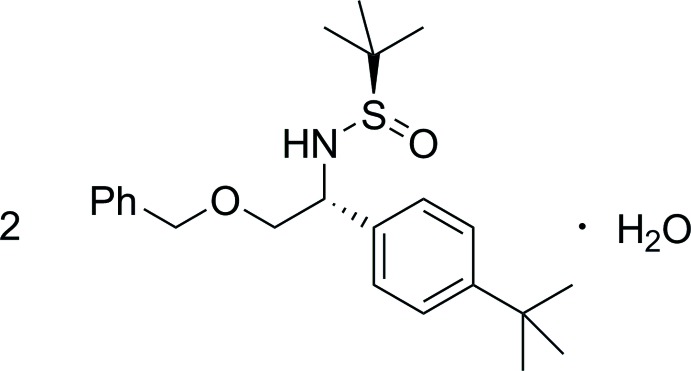


## Experimental   

### 

#### Crystal data  

2C_23_H_33_NO_2_S·H_2_O
*M* *_r_* = 793.14
Monoclinic, 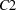

*a* = 21.5717 (17) Å
*b* = 6.1097 (5) Å
*c* = 17.0838 (14) Å
β = 93.2220 (17)°
*V* = 2248.0 (3) Å^3^
*Z* = 2
Mo *K*α radiation
μ = 0.16 mm^−1^
*T* = 100 K
0.40 × 0.18 × 0.16 mm

#### Data collection  

Bruker SMART APEXII CCD platform diffractometer
Absorption correction: multi-scan (*SADABS*; Sheldrick, 2012 ▶) *T*_min_ = 0.662, *T*_max_ = 0.748
34281 measured reflections
12280 independent reflections
9359 reflections with *I* > 2σ(*I*)
*R*_int_ = 0.049

#### Refinement  

*R*[*F* ^2^ > 2σ(*F* ^2^)] = 0.050
*wR*(*F* ^2^) = 0.110
*S* = 1.01
12280 reflections
385 parameters
1 restraint
All H-atom parameters refined
Δρ_max_ = 0.46 e Å^−3^
Δρ_min_ = −0.25 e Å^−3^
Absolute structure: Flack parameter determined using 3436 quotients [(*I* ^+^)−(*I* ^−^)]/[(*I* ^+^)+(*I* ^−^)] (Parsons & Flack, 2004 ▶)
Absolute structure parameter: −0.01 (3)

### 

Data collection: *APEX2* (Bruker, 2013 ▶); cell refinement: *SAINT* (Bruker, 2013 ▶); data reduction: *SAINT*; program(s) used to solve structure: *SIR2011* (Burla *et al.*, 2012 ▶); program(s) used to refine structure: *SHELXL2013* (Sheldrick, 2008 ▶); molecular graphics: *SHELXTL* (Sheldrick, 2008 ▶); software used to prepare material for publication: *SHELXTL*.

## Supplementary Material

Crystal structure: contains datablock(s) I, global. DOI: 10.1107/S1600536814004589/gk2604sup1.cif

Structure factors: contains datablock(s) I. DOI: 10.1107/S1600536814004589/gk2604Isup2.hkl

Supporting information file. DOI: 10.1107/S1600536814004589/gk2604Isup3.cml

CCDC reference: 989104

Additional supporting information:  crystallographic information; 3D view; checkCIF report

## Figures and Tables

**Table 1:** Hydrogen-bond geometry (Å, °)

*D*—H⋯*A*	*D*—H	H⋯*A*	*D*⋯*A*	*D*—H⋯*A*
O3—H3*O*⋯O2	0.82 (3)	2.02 (3)	2.8420 (19)	171 (3)
C2—H2*A*⋯O2^i^	0.96 (2)	2.52 (2)	3.372 (2)	148.1 (19)
C21—H21*C*⋯O3^i^	0.97 (2)	2.44 (2)	3.397 (3)	169.0 (19)

Symmetry code: (i) 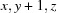.

**Fig. 1.**
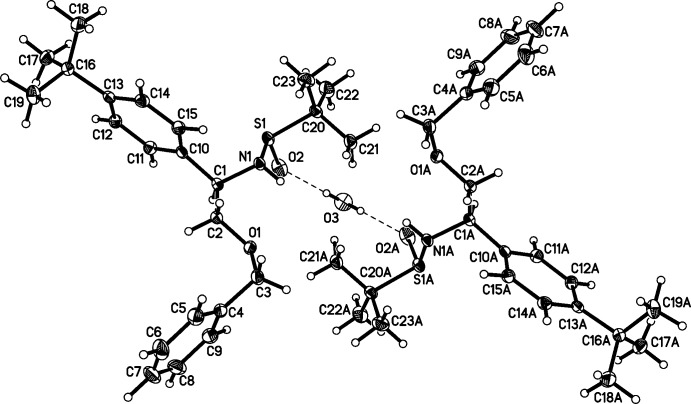
Molecular structure of the title material, showing the atom numbering. The two organic molecules are related by a crystallographic twofold axis (1 - x, y, -z) that includes the water molecule. Intermolecular O–H···O hydrogen bonding is represented with dashed lines. Displacement ellipsoids are drawn at the 50% probability level.

**Fig. 2.**
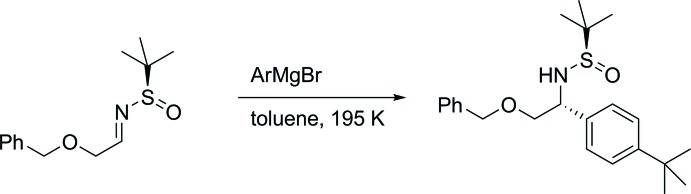
Reaction scheme.

## supplementary crystallographic information

### 1. Comment

We report the synthesis, isolation, and characterization of the protected 1,2-aminoalcohol (*R*)-*N*-((*R*)-2-(benzyloxy)- 1-(4-(*tert*-butyl)phenyl)ethyl)-2-methylpropane-2-sulfinamide (Fig. 1) from the addition of 4-*tert*-butylphenylmagnesium bromide to an *N*-*tert*-butanesulfinyl imine (Fig. 2) according to the procedure of Ellman (Tang *et al.*, 2001). 1,2-Aminoalcohols are found in a variety of pharmaceutically active compounds and are an important component of the chiral pool (Bergmeier, 2000). The method of Ellman and Tang is one of the most direct to monosubstituted aminoalcohols and relies upon the chiral ammonia equivalent, 2-methyl-2-propanesulfinamide (*tert*-butanesulfinamide). In the original report, the absolute configuration of the products was determined by comparison of optical rotations and no structures of the products of these reactions have been reported in the database. This structure is consistent with the sense of induction reported by Ellman and Tang (Tang *et al.*, 2001). There are 21 structures of *N*-sulfinyl-protected 1,2-aminoalcohols in the Cambridge Stuctural Database, but only two of these structures have no substitution at the 1 position and have substitution at the 2 position (Allen, 2002, refcodes FOKDUF, YEQBOM). Neither of these two structures was made by the method we used and neither has an aryl group at the 2 position.

### 2. Experimental

Dry solvents were prepared from ACS grade, inhibitor free solvents by passage through activated molecular sieves in an Innovative Technology solvent purification system. CDCl_3_ was purchased from Cambridge Isotope Laboratories, Inc., dried over molecular sieves, and stored in a desiccator until use. ^1^H and ^13^C NMR spectra were recorded on an Avance 500 MHz s pectrometer with residual protiated solvent as a reference.

To an oven dried 50 ml Schlenk flask equipped with a magnetic stir bar and a rubber septum, 0.685 g (3.9 mmol) of (*R*)-*N*-(2-(benzyloxy)ethylidene)-2-methylpropane-2-sulfinamide, prepared from 2-butene-1,4-diol (Evans *et al.*, 2008, and Tang *et al.*, 2001), and 20 ml of toluene were added, and the mixture was cooled to 195 K under nitrogen (Fig. 2). Also at 195 K and under positive nitrogen pressure, Grignard reagent 4-*tert*-butylmagnesium bromide (1.5 equivalents, 5.85 mmol), prepared from 4-*tert*-butylbromobenzene (Tilstam and Weinmann, 2002), was added dropwise *via* cannula. The reaction was stirred at 195 K until complete consumption of the imine was confirmed by TLC (25% ethyl acetate in hexanes). After quenching with aqueous saturated sodium sulfate, the mixture was warmed to room temperature, dried over sodium sulfate, and filtered through Celite. By ^1^H NMR, the crude product was a 3.6:1 ratio of diastereomers, favoring the title compound. Solvent was removed under reduced pressure and the resultant viscous yellow oil was purified by column chromatography (25% ethyl acetate in hexanes) to yield a white solid (266 mg, 18% yield). Initially single crystals of the unsolvated material were grown from a saturated pentane solution at 243 K. NMR analysis of these crystals showed only the title compound and none of the minor diastereomer, (*R*)-*N*-((*S*)-2-(benzyloxy)-1-(4-(*tert*- butyl)phenyl)ethyl)-2-methylpropane-2-sulfinamide. However, the structure suffered from severe disorder. High quality single crystals of the title material were obtained from slow evaporation of a methanol solution at room temperature.

The following characterizations were performed on the unsolvated material: Mp 334–336 K. ^1^H NMR (CDCl_3_, 500 MHz): δ 7.37–7.26 (m, 9H), 4.69–4.73 (m, 1H), 4.65 (d, *J* = 11 Hz, 1H), 4.54 (d, *J* = 12 Hz, 1H), 4.18 (s, 1H), 3.67–3.57 (m, 2H), 1.31 (s, 9H), 1.23 (s, 9H) p.p.m.. ^13^C{^1^H} NMR (CDCl_3_, 125 MHz): δ 150.90, 137.70, 135.37, 128.47, 128.36, 127.83, 127.54, 125.39, 74.19, 72.67, 56.89, 55.49, 34.55, 31.35, 22.64 p.p.m.. IR (neat): 3306, 1061 cm^-1^.

Crystal data for the highly disordered unsolvated compound: Orthorhombic, *P*2_1_2_1_2_1_; Cell constants (Å, °): *a* = 5.6753 (7), *b* = 17.305 (2), *c* = 22.188 (3); *V* = 2179.2 (5) Å^3^; *Z* = 4; *T* = 100.0 (5) K; 12121 reflections (9622 for [*I* > 2*σ*(*I*)]).

### 3. Refinement

All H atoms were located from difference Fourier maps and freely refined.

### Crystal data

**Table:**

2C_23_H_33_NO_2_S·H_2_O	*F*(000) = 860
*M**_r_* = 793.14	*D*_x_ = 1.172 Mg m^−^^3^
Monoclinic, *C*2	Mo *K*α radiation, λ = 0.71073 Å
*a* = 21.5717 (17) Å	Cell parameters from 4053 reflections
*b* = 6.1097 (5) Å	θ = 2.2–37.9°
*c* = 17.0838 (14) Å	µ = 0.16 mm^−^^1^
β = 93.2220 (17)°	*T* = 100 K
*V* = 2248.0 (3) Å^3^	Needle, colorless
*Z* = 2	0.40 × 0.18 × 0.16 mm

### Data collection

**Table:**

Bruker SMART APEXII CCD platform diffractometer	9359 reflections with *I* > 2σ(*I*)
Radiation source: fine-focus sealed tube	*R*_int_ = 0.049
ω scans	θ_max_ = 38.7°, θ_min_ = 1.9°
Absorption correction: multi-scan (*SADABS*; Sheldrick, 2012)	*h* = −37→37
*T*_min_ = 0.662, *T*_max_ = 0.748	*k* = −10→10
34281 measured reflections	*l* = −29→29
12280 independent reflections	

### Refinement

**Table:**

Refinement on *F*^2^	Secondary atom site location: difference Fourier map
Least-squares matrix: full	Hydrogen site location: difference Fourier map
*R*[*F*^2^ > 2σ(*F*^2^)] = 0.050	All H-atom parameters refined
*wR*(*F*^2^) = 0.110	*w* = 1/[σ^2^(*F*_o_^2^) + (0.0513*P*)^2^] where *P* = (*F*_o_^2^ + 2*F*_c_^2^)/3
*S* = 1.01	(Δ/σ)_max_ = 0.001
12280 reflections	Δρ_max_ = 0.46 e Å^−^^3^
385 parameters	Δρ_min_ = −0.25 e Å^−^^3^
1 restraint	Absolute structure: Flack parameter determined using 3436 quotients [(*I*^+^)-(*I*^-^)]/[(*I*^+^)+(*I*^-^)] (Parsons & Flack, 2004)
Primary atom site location: structure-invariant direct methods	Absolute structure parameter: −0.01 (3)

### Special details

**Table:**

Geometry. All e.s.d.'s (except the e.s.d. in the dihedral angle between two l.s. planes) are estimated using the full covariance matrix. The cell e.s.d.'s are taken into account individually in the estimation of e.s.d.'s in distances, angles and torsion angles; correlations between e.s.d.'s in cell parameters are only used when they are defined by crystal symmetry. An approximate (isotropic) treatment of cell e.s.d.'s is used for estimating e.s.d.'s involving l.s. planes.
Refinement. All hydrogen atoms were found from the difference Fourier map and refined freely.The absolute configuration was deterimined using 3436 quotients, which gave a Flack parameter of -0.01 (3) (Parsons and Flack, 2004, Parson *et al.*, 2013). This is essentially the same value obtained without *D*_obs_(h) as a restraint, which resulted in a Flack parameter of -0.01 (4), calculated from 5617 Friedel pairs (Flack, 1983).

### Fractional atomic coordinates and isotropic or equivalent isotropic displacement parameters (Å^2^)

**Table:**

	*x*	*y*	*z*	*U*_iso_*/*U*_eq_	
S1	0.42226 (2)	0.46312 (7)	0.15482 (2)	0.01727 (8)	
O1	0.58554 (6)	0.8370 (2)	0.16151 (7)	0.0209 (2)	
O2	0.46240 (7)	0.2778 (2)	0.13077 (8)	0.0285 (3)	
N1	0.46287 (6)	0.6943 (2)	0.16175 (8)	0.0163 (2)	
H1N	0.4839 (11)	0.719 (4)	0.1199 (14)	0.030 (6)*	
C1	0.50207 (7)	0.7109 (3)	0.23545 (9)	0.0161 (3)	
H1	0.5262 (10)	0.574 (4)	0.2460 (12)	0.016 (5)*	
C2	0.54948 (7)	0.8923 (3)	0.22575 (9)	0.0178 (3)	
H2A	0.5281 (10)	1.029 (4)	0.2189 (14)	0.023 (6)*	
H2B	0.5749 (9)	0.904 (3)	0.2734 (12)	0.012 (5)*	
C3	0.62305 (8)	1.0173 (3)	0.13942 (10)	0.0227 (3)	
H3A	0.5959 (11)	1.147 (4)	0.1336 (13)	0.022 (6)*	
H3B	0.6351 (9)	0.979 (4)	0.0868 (13)	0.022 (5)*	
C4	0.67655 (8)	1.0589 (3)	0.19842 (9)	0.0197 (3)	
C5	0.68504 (9)	1.2612 (3)	0.23440 (11)	0.0255 (4)	
H5	0.6574 (12)	1.379 (5)	0.2226 (15)	0.037 (7)*	
C6	0.73526 (11)	1.2977 (4)	0.28735 (12)	0.0341 (5)	
H6	0.7411 (11)	1.431 (5)	0.3100 (15)	0.036 (7)*	
C7	0.77729 (10)	1.1315 (4)	0.30506 (13)	0.0349 (5)	
H7	0.8120 (12)	1.157 (5)	0.3420 (15)	0.041 (7)*	
C8	0.76889 (9)	0.9273 (4)	0.27026 (12)	0.0314 (5)	
H8	0.7981 (13)	0.816 (5)	0.2848 (16)	0.046 (8)*	
C9	0.71873 (8)	0.8916 (3)	0.21733 (11)	0.0242 (3)	
H9	0.7099 (12)	0.759 (5)	0.1917 (14)	0.033 (7)*	
C10	0.46395 (7)	0.7656 (3)	0.30471 (8)	0.0154 (3)	
C11	0.46392 (7)	0.6297 (3)	0.36989 (10)	0.0177 (3)	
H11	0.4859 (10)	0.499 (4)	0.3673 (13)	0.024 (6)*	
C12	0.43313 (8)	0.6904 (3)	0.43609 (9)	0.0181 (3)	
H12	0.4333 (10)	0.594 (4)	0.4788 (13)	0.025 (6)*	
C13	0.40197 (7)	0.8894 (3)	0.44018 (9)	0.0161 (3)	
C14	0.40172 (7)	1.0238 (3)	0.37349 (10)	0.0184 (3)	
H14	0.3801 (11)	1.162 (4)	0.3734 (13)	0.024 (6)*	
C15	0.43132 (7)	0.9623 (3)	0.30689 (9)	0.0185 (3)	
H15	0.4295 (10)	1.052 (4)	0.2600 (13)	0.024 (6)*	
C16	0.37205 (7)	0.9653 (4)	0.51461 (9)	0.0192 (3)	
C17	0.35710 (9)	0.7726 (4)	0.56789 (11)	0.0248 (4)	
H17A	0.3367 (10)	0.810 (4)	0.6113 (13)	0.025 (6)*	
H17B	0.3945 (12)	0.696 (5)	0.5893 (14)	0.034 (7)*	
H17C	0.3302 (12)	0.670 (5)	0.5370 (15)	0.039 (7)*	
C18	0.31087 (10)	1.0872 (5)	0.49419 (13)	0.0340 (5)	
H18A	0.2904 (12)	1.139 (5)	0.5422 (15)	0.038 (7)*	
H18B	0.2843 (13)	1.004 (5)	0.4603 (18)	0.051 (8)*	
H18C	0.3177 (11)	1.213 (5)	0.4672 (15)	0.032 (7)*	
C19	0.41775 (9)	1.1181 (3)	0.55980 (11)	0.0249 (3)	
H19A	0.4012 (10)	1.170 (4)	0.6085 (13)	0.020 (5)*	
H19B	0.4268 (10)	1.243 (4)	0.5268 (12)	0.018 (5)*	
H19C	0.4586 (9)	1.037 (4)	0.5743 (12)	0.017 (5)*	
C20	0.37165 (7)	0.5317 (3)	0.06805 (10)	0.0182 (3)	
C21	0.40958 (8)	0.5784 (3)	−0.00242 (10)	0.0215 (3)	
H21A	0.3833 (11)	0.591 (4)	−0.0478 (14)	0.026 (6)*	
H21B	0.4409 (10)	0.450 (5)	−0.0104 (14)	0.030 (6)*	
H21C	0.4312 (11)	0.717 (4)	0.0028 (13)	0.023 (6)*	
C22	0.33246 (10)	0.3261 (4)	0.05314 (12)	0.0293 (4)	
H22A	0.3027 (14)	0.348 (6)	0.0101 (18)	0.052 (8)*	
H22B	0.3099 (12)	0.280 (5)	0.0976 (15)	0.030 (6)*	
H22C	0.3551 (13)	0.198 (5)	0.0423 (16)	0.049 (8)*	
C23	0.33169 (9)	0.7248 (4)	0.09006 (12)	0.0269 (4)	
H23A	0.2998 (13)	0.757 (5)	0.0466 (16)	0.047 (8)*	
H23B	0.3557 (11)	0.851 (4)	0.0981 (14)	0.029 (6)*	
H23C	0.3068 (11)	0.692 (4)	0.1352 (14)	0.028 (6)*	
O3	0.5000	0.0337 (4)	0.0000	0.0323 (4)	
H3O	0.4903 (13)	0.116 (5)	0.0355 (16)	0.047 (9)*	

### Atomic displacement parameters (Å^2^)

**Table:**

	*U*^11^	*U*^22^	*U*^33^	*U*^12^	*U*^13^	*U*^23^
S1	0.01922 (16)	0.01591 (15)	0.01623 (15)	−0.00308 (15)	−0.00284 (12)	0.00315 (15)
O1	0.0192 (5)	0.0285 (6)	0.0150 (5)	−0.0090 (5)	0.0021 (4)	−0.0026 (4)
O2	0.0358 (7)	0.0181 (6)	0.0304 (7)	0.0067 (5)	−0.0080 (6)	0.0006 (5)
N1	0.0162 (6)	0.0182 (6)	0.0140 (5)	−0.0052 (5)	−0.0017 (4)	0.0013 (5)
C1	0.0150 (6)	0.0194 (7)	0.0137 (6)	0.0000 (5)	−0.0018 (5)	0.0007 (5)
C2	0.0153 (6)	0.0233 (7)	0.0146 (6)	−0.0031 (6)	−0.0005 (5)	−0.0011 (6)
C3	0.0225 (7)	0.0301 (9)	0.0152 (7)	−0.0099 (6)	−0.0015 (6)	0.0035 (6)
C4	0.0181 (7)	0.0251 (8)	0.0157 (6)	−0.0066 (6)	0.0011 (5)	0.0007 (6)
C5	0.0281 (9)	0.0244 (9)	0.0241 (8)	−0.0056 (7)	0.0014 (7)	−0.0009 (7)
C6	0.0412 (11)	0.0354 (11)	0.0253 (9)	−0.0187 (9)	−0.0020 (8)	−0.0050 (8)
C7	0.0265 (9)	0.0502 (13)	0.0267 (9)	−0.0176 (9)	−0.0092 (7)	0.0087 (9)
C8	0.0203 (7)	0.0418 (13)	0.0316 (9)	−0.0043 (8)	−0.0033 (7)	0.0136 (9)
C9	0.0235 (8)	0.0252 (8)	0.0241 (8)	−0.0047 (7)	0.0024 (6)	0.0027 (7)
C10	0.0139 (6)	0.0184 (7)	0.0134 (6)	−0.0015 (5)	−0.0025 (5)	0.0013 (5)
C11	0.0189 (7)	0.0162 (7)	0.0178 (7)	0.0006 (6)	0.0005 (5)	0.0022 (5)
C12	0.0192 (7)	0.0194 (7)	0.0156 (6)	0.0007 (6)	0.0002 (5)	0.0033 (5)
C13	0.0132 (6)	0.0198 (7)	0.0149 (6)	−0.0014 (5)	−0.0023 (5)	0.0000 (5)
C14	0.0168 (6)	0.0184 (7)	0.0197 (7)	0.0030 (5)	−0.0004 (5)	0.0029 (5)
C15	0.0179 (6)	0.0210 (6)	0.0166 (6)	0.0020 (7)	0.0001 (5)	0.0050 (7)
C16	0.0175 (6)	0.0245 (7)	0.0156 (6)	0.0022 (7)	−0.0007 (5)	−0.0018 (7)
C17	0.0237 (8)	0.0308 (9)	0.0205 (8)	−0.0048 (7)	0.0062 (6)	−0.0014 (7)
C18	0.0269 (9)	0.0531 (15)	0.0220 (9)	0.0182 (9)	0.0004 (7)	−0.0013 (9)
C19	0.0328 (9)	0.0221 (8)	0.0195 (8)	−0.0039 (7)	−0.0018 (7)	−0.0022 (6)
C20	0.0157 (6)	0.0195 (7)	0.0189 (7)	−0.0039 (5)	−0.0030 (5)	0.0023 (5)
C21	0.0233 (7)	0.0233 (8)	0.0174 (7)	−0.0027 (6)	−0.0024 (6)	0.0046 (6)
C22	0.0283 (9)	0.0320 (10)	0.0270 (9)	−0.0142 (8)	−0.0033 (7)	−0.0004 (8)
C23	0.0206 (8)	0.0292 (10)	0.0307 (9)	0.0044 (7)	−0.0012 (7)	0.0029 (8)
O3	0.0374 (11)	0.0194 (9)	0.0399 (12)	0.000	0.0008 (10)	0.000

### Geometric parameters (Å, º)

**Table:**

S1—O2	1.4967 (14)	C12—H12	0.94 (2)
S1—N1	1.6628 (14)	C13—C14	1.404 (2)
S1—C20	1.8394 (16)	C13—C16	1.530 (2)
O1—C2	1.421 (2)	C14—C15	1.387 (2)
O1—C3	1.430 (2)	C14—H14	0.96 (2)
N1—C1	1.480 (2)	C15—H15	0.97 (2)
N1—H1N	0.88 (2)	C16—C17	1.533 (3)
C1—C10	1.516 (2)	C16—C19	1.534 (3)
C1—C2	1.523 (2)	C16—C18	1.538 (3)
C1—H1	1.00 (2)	C17—H17A	0.91 (2)
C2—H2A	0.96 (2)	C17—H17B	0.99 (3)
C2—H2B	0.96 (2)	C17—H17C	0.99 (3)
C3—C4	1.511 (2)	C18—H18A	1.00 (3)
C3—H3A	0.99 (2)	C18—H18B	0.94 (3)
C3—H3B	0.98 (2)	C18—H18C	0.91 (3)
C4—C5	1.388 (3)	C19—H19A	0.98 (2)
C4—C9	1.394 (3)	C19—H19B	0.97 (2)
C5—C6	1.390 (3)	C19—H19C	1.03 (2)
C5—H5	0.95 (3)	C20—C21	1.520 (2)
C6—C7	1.383 (4)	C20—C23	1.521 (3)
C6—H6	0.91 (3)	C20—C22	1.527 (3)
C7—C8	1.390 (4)	C21—H21A	0.94 (2)
C7—H7	0.96 (3)	C21—H21B	1.05 (3)
C8—C9	1.388 (3)	C21—H21C	0.97 (2)
C8—H8	0.95 (3)	C22—H22A	0.96 (3)
C9—H9	0.94 (3)	C22—H22B	0.97 (3)
C10—C11	1.389 (2)	C22—H22C	0.94 (3)
C10—C15	1.394 (2)	C23—H23A	1.00 (3)
C11—C12	1.394 (2)	C23—H23B	0.94 (3)
C11—H11	0.93 (2)	C23—H23C	0.99 (2)
C12—C13	1.393 (2)	O3—H3O	0.82 (3)
			
O2—S1—N1	110.59 (8)	C15—C14—C13	121.74 (16)
O2—S1—C20	106.10 (8)	C15—C14—H14	118.7 (14)
N1—S1—C20	98.65 (7)	C13—C14—H14	119.5 (14)
C2—O1—C3	111.30 (14)	C14—C15—C10	120.77 (15)
C1—N1—S1	113.10 (11)	C14—C15—H15	121.8 (13)
C1—N1—H1N	112.5 (16)	C10—C15—H15	117.4 (13)
S1—N1—H1N	112.5 (17)	C13—C16—C17	111.92 (16)
N1—C1—C10	111.72 (12)	C13—C16—C19	108.26 (13)
N1—C1—C2	108.20 (13)	C17—C16—C19	108.61 (14)
C10—C1—C2	108.77 (13)	C13—C16—C18	110.78 (13)
N1—C1—H1	111.3 (12)	C17—C16—C18	107.46 (16)
C10—C1—H1	110.2 (12)	C19—C16—C18	109.78 (18)
C2—C1—H1	106.4 (12)	C16—C17—H17A	114.7 (15)
O1—C2—C1	108.14 (13)	C16—C17—H17B	113.0 (15)
O1—C2—H2A	113.2 (14)	H17A—C17—H17B	103.8 (19)
C1—C2—H2A	109.1 (13)	C16—C17—H17C	107.9 (16)
O1—C2—H2B	111.2 (11)	H17A—C17—H17C	107 (2)
C1—C2—H2B	108.4 (12)	H17B—C17—H17C	110 (2)
H2A—C2—H2B	106.8 (19)	C16—C18—H18A	112.2 (15)
O1—C3—C4	112.08 (14)	C16—C18—H18B	111.4 (19)
O1—C3—H3A	107.7 (13)	H18A—C18—H18B	113 (2)
C4—C3—H3A	111.0 (14)	C16—C18—H18C	111.1 (16)
O1—C3—H3B	103.9 (14)	H18A—C18—H18C	104 (2)
C4—C3—H3B	114.9 (12)	H18B—C18—H18C	105 (2)
H3A—C3—H3B	106.7 (18)	C16—C19—H19A	111.7 (13)
C5—C4—C9	118.82 (17)	C16—C19—H19B	109.2 (13)
C5—C4—C3	121.48 (17)	H19A—C19—H19B	109.6 (18)
C9—C4—C3	119.70 (17)	C16—C19—H19C	110.3 (12)
C4—C5—C6	120.6 (2)	H19A—C19—H19C	107.4 (18)
C4—C5—H5	121.0 (16)	H19B—C19—H19C	108.5 (17)
C6—C5—H5	118.3 (17)	C21—C20—C23	112.84 (15)
C7—C6—C5	120.1 (2)	C21—C20—C22	109.86 (15)
C7—C6—H6	119.5 (16)	C23—C20—C22	111.29 (15)
C5—C6—H6	120.3 (16)	C21—C20—S1	111.06 (11)
C6—C7—C8	119.82 (19)	C23—C20—S1	107.24 (12)
C6—C7—H7	120.2 (17)	C22—C20—S1	104.18 (12)
C8—C7—H7	120.0 (17)	C20—C21—H21A	110.1 (14)
C9—C8—C7	119.9 (2)	C20—C21—H21B	110.0 (13)
C9—C8—H8	122.6 (18)	H21A—C21—H21B	109 (2)
C7—C8—H8	117.6 (18)	C20—C21—H21C	111.6 (13)
C8—C9—C4	120.71 (19)	H21A—C21—H21C	106 (2)
C8—C9—H9	124.8 (16)	H21B—C21—H21C	110.8 (19)
C4—C9—H9	114.5 (16)	C20—C22—H22A	111 (2)
C11—C10—C15	118.08 (14)	C20—C22—H22B	114.0 (16)
C11—C10—C1	121.25 (15)	H22A—C22—H22B	107 (2)
C15—C10—C1	120.51 (13)	C20—C22—H22C	115.1 (18)
C10—C11—C12	120.89 (15)	H22A—C22—H22C	107 (3)
C10—C11—H11	116.9 (14)	H22B—C22—H22C	102 (2)
C12—C11—H11	122.2 (14)	C20—C23—H23A	110.2 (17)
C13—C12—C11	121.75 (15)	C20—C23—H23B	111.1 (15)
C13—C12—H12	119.3 (14)	H23A—C23—H23B	107 (2)
C11—C12—H12	118.9 (14)	C20—C23—H23C	112.1 (15)
C12—C13—C14	116.72 (14)	H23A—C23—H23C	104 (2)
C12—C13—C16	122.24 (15)	H23B—C23—H23C	112 (2)
C14—C13—C16	120.98 (15)		
			
O2—S1—N1—C1	−79.01 (13)	C15—C10—C11—C12	−1.2 (2)
C20—S1—N1—C1	170.09 (11)	C1—C10—C11—C12	174.35 (15)
S1—N1—C1—C10	−75.83 (16)	C10—C11—C12—C13	−0.8 (3)
S1—N1—C1—C2	164.47 (11)	C11—C12—C13—C14	1.6 (2)
C3—O1—C2—C1	169.06 (13)	C11—C12—C13—C16	−175.44 (15)
N1—C1—C2—O1	−59.44 (17)	C12—C13—C14—C15	−0.4 (2)
C10—C1—C2—O1	179.02 (13)	C16—C13—C14—C15	176.72 (15)
C2—O1—C3—C4	72.42 (18)	C13—C14—C15—C10	−1.7 (3)
O1—C3—C4—C5	−123.42 (18)	C11—C10—C15—C14	2.4 (2)
O1—C3—C4—C9	56.8 (2)	C1—C10—C15—C14	−173.17 (15)
C9—C4—C5—C6	1.1 (3)	C12—C13—C16—C17	−23.2 (2)
C3—C4—C5—C6	−178.68 (17)	C14—C13—C16—C17	159.92 (15)
C4—C5—C6—C7	−0.3 (3)	C12—C13—C16—C19	96.5 (2)
C5—C6—C7—C8	−0.5 (3)	C14—C13—C16—C19	−80.41 (19)
C6—C7—C8—C9	0.6 (3)	C12—C13—C16—C18	−143.07 (19)
C7—C8—C9—C4	0.1 (3)	C14—C13—C16—C18	40.0 (2)
C5—C4—C9—C8	−1.0 (3)	O2—S1—C20—C21	−54.50 (14)
C3—C4—C9—C8	178.79 (16)	N1—S1—C20—C21	59.96 (13)
N1—C1—C10—C11	122.18 (16)	O2—S1—C20—C23	−178.21 (12)
C2—C1—C10—C11	−118.45 (17)	N1—S1—C20—C23	−63.75 (13)
N1—C1—C10—C15	−62.37 (19)	O2—S1—C20—C22	63.72 (14)
C2—C1—C10—C15	56.99 (18)	N1—S1—C20—C22	178.18 (13)

### Hydrogen-bond geometry (Å, º)

**Table:**

*D*—H···*A*	*D*—H	H···*A*	*D*···*A*	*D*—H···*A*
O3—H3*O*···O2	0.82 (3)	2.02 (3)	2.8420 (19)	171 (3)
C2—H2*A*···O2^i^	0.96 (2)	2.52 (2)	3.372 (2)	148.1 (19)
C21—H21*C*···O3^i^	0.97 (2)	2.44 (2)	3.397 (3)	169.0 (19)

Symmetry code: (i) *x*, *y*+1, *z*.

## References

[bb1] Allen, F. H. (2002). *Acta Cryst.* B**58**, 380–388. 10.1107/s0108768102003890 12037359

[bb2] Bergmeier, S. C. (2000). *Tetrahedron*, **56**, 2561–2576.

[bb3] Bruker (2013). *APEX2* and *SAINT* Bruker AXS, Inc., Madison, WI, USA.

[bb4] Burla, M. C., Caliandro, R., Camalli, M., Carrozzini, B., Cascarano, G. L., Giacovazzo, C., Mallamo, M., Mazzone, A., Polidori, G. & Spagna, R. (2012). *J. Appl. Cryst.* **45**, 357–361.

[bb5] Evans, D. A., Kvaernø, L., Dunn, T. B., Beauchemin, A., Raymer, B., Mulder, J. A., Olhava, E. J., Juhl, M., Kagechika, K. & Favor, D. A. (2008). *J. Am. Chem. Soc.* **130**, 16295–16309. 10.1021/ja804659n PMC3408805 19006391

[bb6] Flack, H. D. (1983). *Acta Cryst.* A**39**, 876–881.

[bb7] Parsons, S. & Flack, H. (2004). *Acta Cryst.* A**60**, s61.

[bb8] Parsons, S., Flack, H. D. & Wagner, T. (2013). *Acta Cryst.* B**69**, 249–259. 10.1107/S2052519213010014 PMC3661305 23719469

[bb9] Sheldrick, G. M. (2008). *Acta Cryst.* A**64**, 112–122. 10.1107/S0108767307043930 18156677

[bb10] Sheldrick, G. M. (2012). *SADABS* University of Göttingen, Germany.

[bb11] Tang, T. P., Volkman, S. K. & Ellman, J. A. (2001). *J. Org. Chem.* **66**, 8772–8778. 10.1021/jo0156868 11749605

[bb12] Tilstam, U. & Weinmann, H. (2002). *Org. Process Res. Dev.* **6**, 906–910.

